# Effects of acceptance and commitment therapy on self-management skills and psychological resilience of young and middle-aged patients underwent percutaneous transluminal coronary intervention for primary myocardial infarction: a pilot study

**DOI:** 10.1186/s13063-021-05923-0

**Published:** 2022-01-12

**Authors:** Jiaoyu Cao, Panpan Sun, Lixiang Zhang, Xia Chen, Wenjuan Gui, Anping Ou, Kaibing Chen, Likun Ma

**Affiliations:** 1 Department of Cardiology, The First Affiliated Hospital of University of Science and Technology of China (Anhui Provincial Hospital), Hefei, China; 2 Department of Cardiovascular Surgery, The First Affiliated Hospital of University of Science and Technology of China (Anhui Provincial Hospital), Hefei, China; 3 Department of Nursing, The First Affiliated Hospital of University of Science and Technology of China (Anhui Provincial Hospital), Hefei, China

**Keywords:** Acceptance and commitment therapy, Myocardial infarction, Percutaneous coronary intervention, Physical and psychological health, Psychotherapy

## Abstract

**Background:**

Acceptance and commitment therapy (ACT) is an intervention focusing on altering how patients relate to their thoughts. This study aimed to investigate the effects of ACT on self-management ability and psychological resilience of young and middle-aged patients undergoing percutaneous transluminal coronary intervention (PCI) for primary myocardial infarction (MI).

**Methods:**

This pilot study included 98 young and middle-aged patients who underwent PCI for primary MI using a convenient sampling method. The patients were divided into a control group and an ACT group using the random number table method. The patients in the control group received routine nursing, while those in the ACT group received routine nursing combined with ACT.

**Results:**

The psychological resilience and self-management ability scores were significantly higher in the ACT group than in the control group 3 months after the intervention (*P* < 0.001 and < 0.05, respectively). In addition, compared to the baseline scores of psychological resilience and self-management ability, these scores were significantly higher in the ACT group at 3 months post-intervention (*P* < 0.001 and < 0.05, respectively).

**Conclusion:**

ACT could enhance the psychological resilience and self-efficacy and improve the self-management ability of young and middle-aged patients who underwent PCI for primary MI.

**Trial registration:**

China Clinical Trial Center ChiCTR2000029775. Registered on 13 February 2020. Registration title:Study on the popularization and application of rotational atherectomy for the treatment of severely calcified coronary lesions.

## Background

Acute myocardial infarction (AMI) is a common potentially lethal heart disease characterized by myocardial necrosis induced by acute and persistent ischemia and anoxia of a coronary artery [1]. Percutaneous transluminal coronary intervention (PCI) is the most common intervention for AMI and is known to effectively improve the blood and oxygen supply to the myocardium [2]. Traditionally identified risk factors related to lifestyle choices, such as smoking, male gender, and family history of cardiovascular heart disease, are associated with the incidence of AMI in young patients. A large study on a national US database from 2001 to 2010 revealed that the hospitalization rates for young patients with AMI did not decline over time [3].

Many studies have shown that anxiety is significantly higher in young and middle-aged patients than in elderly patients, and the consequent decrease of the quality of life and early loss of working capability could bring heavy social, financial, and psychological burdens [3, 4]. Due to the different degrees of physical and psychological issues, more and more young and middle-aged AMI patients display reduced self-efficiency and treatment compliance, which substantially influence the disease outcomes [5]. Therefore, it is important to apply effective psychological interventions to such patients.

Acceptance and commitment therapy (ACT) is an action-based approach that originates from cognitive behavior therapy. Since its introduction in 1982 by Steven C. Hayes, ACT has been effectively used to help patients accept conditions that are beyond their control and commit to actions that can enrich their lives [6]. ACT is based on the concept that suffering is a natural and inevitable human condition. Though humans have an instinct to control their experiences, this instinct does not always serve them. ACT has been successfully applied to treat workplace stress, test anxiety, social anxiety disorder, depression, obsessive-compulsive disorder, and psychosis. In addition to treating mental health conditions, ACT has also been reported to successfully treat a range of medical conditions. As of August 2016 [7], a total of 136 RCTs of ACT have been published, including psychiatric (e.g., depression, anxiety, psychosis, substance abuse, and eating disorders), medical (e.g., chronic pain, cancer, epilepsy, and diabetes), behavioral (e.g., smoking and weight loss), and other disorders and issues (e.g., stigma, parenting, chess performance, work performance, and human prejudice). The earliest meta-analysis on this subject was published in 2009 and demonstrated that ACT was more effective than placebo or conventional treatments to improve most psychological issues (except for anxiety and depression) [8]. Currently, ACT has been used as an additional treatment for psychological interventions in adult patients with long-term chronic pain or other chronic diseases, aiming to release their distress. Still, most previous studies involved patients of 18–75 years of age. Thus far, no ACT study has focused on young and middle-aged patients.

Therefore, in the present study, we aimed to investigate the effects of ACT on the psychological health of young and middle-aged patients with primary MI who received PCI treatment and assess the treatment’s clinical application value.

## Methods

### Study design and participants

It was a randomized, controlled pilot study that focused on the effects of ACT on the psychological health of young and middle-aged patients who underwent PCI for primary MI. This study was approved by the Ethics Committee of the First Affiliated Hospital of the University of Science and Technology in China [KF2018007]. All patients consented to participate in the study by signing the informed consent form.

### Inclusion and exclusion criteria

This prospective study included young and middle-aged patients (18–60 years) with AMI who underwent PCI in our hospital between February 2019 and September 2019. The inclusion criteria were (1) patients diagnosed with AMI according to the 2001 revised criteria of the Cardiovascular Medicine Branch of the Chinese Medical Association and had undergone PCI, (2) the patients were conscious and able to communicate clearly, (3) the patients were 18–60 years, (4) the patients consented to participate in the study by signing the informed consent form, and (5) the patients were able to complete the questionnaire.

The exclusion criteria were (1) patients with severe systemic infection, severe anemia, or cachexia; (2) patients who currently or previously had psychological disorders; or (3) those who have previously undergone PCI for ≥ 2 times.

### Randomization and blinding

Patients were collected by the convenient sampling method and divided into a control group and an ACT group using the random number table method. The interventions were performed by nurses according to the grouping results, while the patients were blinded to the intervention methods. The results of the questionnaire were randomly processed and analyzed by a masked specialist.

### Interventions

All patients received the appropriate medical treatments and nursing after hospitalization as required to address their disease conditions. The patients in both the control and ACT groups (*n* = 49/group) received conventional nursing, including (1) patients who were informed of disease-related treatment and nursing measures, which could help increase the cooperation of the patients to the treatment; (2) psychological intervention was provided to establish harmonious and pleasant nurse-patient relationships, to encourage the patients to express their feelings, provide specific communications actively, and dredge up emotions, as well as provide positive samples to enhance the confidence of rehabilitation among the patients; (3) a good environment was provided, and nurses ensured that patients had sufficient rest; (4) patients received low-flow oxygen therapy to improve the manifestations of myocardial ischemia and anoxia; (5) standard drug therapy was provided, and the patients received guidance to perform on-bed activities; and (6) systemic health education was provided to all the patients.

In addition to the abovementioned conventional nursing measures, the patients in the ACT group received ACT intervention after receiving the conventional nursing measures. An ACT team was established, including one chief nurse (team leader), two senior nurses, one health manager, and one psychological consultant. The team systemically studied ACT treatments and developed a standardized intervention approach.

We assessed the characteristics of the young and middle-aged patients with AMI at three different stages of physical and psychological adaptation and then administered specific interventions. All patients were administered on the day of admission, 2–3 days after admission, on the day of discharge, and 1 week after discharge, with each intervention lasting 30 min. Table 1 outlines the details of the intervention: (1) confusion and reflection: it is carried out on the day of admission; (2) adaptation and reconstruction: acceptance and cognitive dissociation, 2–3 days after admission; (3) acceptance and integration: observe observation and commitment action, on the day of discharge; and (4) the fourth course of intervention at 1 week after discharge.

**Table 1:** Phases of physical and psychological adaptation, as well as the characteristics and interventions of the six stages of ACT

Stages of physical and psychological adaptation	ACT stage	ACT stage characteristics	Interventions applied
Confusion and reflection	Acceptance	Guide the patients to accept the fact of disease; perceive and face the disease instead of avoiding it	1) Establish a harmonic nurse-patient relationship 2) Explain the disease conditions, listen to patient requirements, and evaluate their psychosocial responses 3) Encourage the patients to express their feelings, inform them of the importance of active cooperation with treatment, and encourage them to accept treatment with an optimistic attitude
Adaptation and reconstruction	Cognitive dissociation	Guide the patients to objectively and calmly recognize issues, negative feelings, and self-thoughts	1) Provide active psychological dredging, enhance communication, promote experience sharing, and guide the patients to identify the most suitable method 2) Actively evaluate patient changes, dissociate them from thoughts and anticipations, and encourage them to face various psychological activities objectively
	Present consciousness	Guide the patients to intentionally pay attention to the present psychological activities, physiological feelings, and external environment	1) Listen to the complaints of patients, provide approval, encourage, and support 2) Empathize with patients in their disease experience, encourage them to pay intentional attention to the current environment and their psychological status, and guide them to view life with a positive attitude, establish their value, and enhance confidence 3) Encourage the patients to believe and accept the medical arrangements without overthinking 4) Guide the patients to exempt themselves from anxieties about the disease by mindful decompression, mind emptying, book and newspaper reading, and music listening, and clarify the current status
	Situational analysis	Guide the patients to objectively experience the changes of physical feelings, character, emotion, and behaviors and establish the self-based perception	1) Positively communicate with the patients and guide them to objectively describe their own experience of the changes of behaviors, emotions, characters, and feelings 2) Help the patients to recall previous successful experiences of overcoming difficulties and help them understand the actual and conceptual selves through various metaphorical expressions 3) Analyze the social characters of the patients and guide them to understand their importance to society and their families
Acceptance and integration	Clearing value perspective	To guide patients to actively seek and identify the direction and value of their future life	To communicate with patients through various methods, including writings, pictures, and videos; to encourage patients to be hopeful about their future; to help them resolve confusion about life; and to help them clarify their life values
	Committed actions	To decide the short- and long-term objectives with the patients and achieve changes and growth	1) Select the appropriate attitude to life and lifestyle based on the long- and short-term life goals 2) Actively work towards the goals, identify personal values, and participate in society with an optimistic and proactive attitude
	Evaluation and intervention after hospital discharge	Reality feelings, recognize themselves, constantly adjust, and affirm the self-value	1) To evaluate the psychological symptoms after discharge, whether there are obvious emotional fluctuations, whether there are psychological collisions in the face of the real world, and whether to take effective measures to relieve them after the stimulation of some adverse events 2) Based on the evaluation of the positive psychological intervention, encourage patients to express the real-life experience actively, guide it positively and effectively, encourage families to actively cooperate at this stage, make the patients cognitive self, accept self, correct bad emotions, and affirm self-worth, with positive attitude return to society and return to work

### Outcomes

In order to assess the effects of ACT on young and middle-aged AMI patients treated with PCI, the primary outcome of this study was the psychological resilience score, and the secondary outcome was self-management skills.

### Data collection and definition

A general data collection questionnaire was used on the day of PCI to collect the general data of the patients. The questionnaire was given out by specifically assigned investigators. The data included sex, age, educational level, income, and treatment regimen. In addition, all the patients were invited to join a WeChat group, in which the QR code of Sojump was updated regularly. The patients could scan the QR code of Sojump to complete the questionnaire to report the evaluation of psychological resilience and self-management skills. All the patients were followed up by phone for 3 months after the intervention to evaluate the efficacy of the intervention, and the patients were asked to scan the QR code of Sojump to complete the questionnaire to evaluate their psychological resilience and self-management skills. All selected patients received data collection for 3 months, and the data collection was complete.

The questionnaire tested for the following indicators. (1) Psychological resilience: this indicator was evaluated using the Psychological Resilience Scale [9], containing 37 items in five dimensions. All the items were assessed on a 7-point Likert scale, and each item was scored from 1 to 7. The total possible score of the scale ranged from 37 to 259 points. A higher score was reflective of higher psychological resilience. The internal consistency reliability of the scale is 0.67–0.9, and the scale is suitable for adults. The Cronbach’s *α* value of the scale is 0.851. Exploratory factor analysis revealed that the KMO of the scale is 0.838, and the Bartlett test showed that the approximate chi-square value is 194.131, the degree of freedom is 64, *P* < 0.001, and the cumulative variance contribution rate is 76.223%, suggesting that this scale has relatively high validity and reliability. (2) Self-management skills: this indicator was evaluated using the Rating Scale of Health Self-management Skills for Adults, with relatively high validity and reliability [10]. This scale comprises a total of seven dimensions and 38 items. Each item was scored on a 5-point Likert scale (1–5 points). Higher scores were reflective of better health self-management skills. The Cronbach’s *α* of this scale is 0.826. Exploratory factor analysis shows the KMO of the scale is 0.823; the Bartlett test shows the approximate chi-square value is 187.018, the degree of freedom is 62, *P* < 0.001, and the cumulative variance contribution rate is 75.775%, suggesting that this scale has relatively high validity and reliability

### Statistical analysis

Statistical Package for Social Sciences (SPSS version 22.0; IBM Corp., Armonk, NY) was used for the statistical analysis. Normally distributed continuous variables were described as mean and standard divisions. An independent *t*-test was used to compare the two groups, and the paired *t*-test was used to compare the pre- and post-intervention values. Categorical variables were compared using the *χ*^2^ test or Fisher’s exact test, as appropriate. A *P* value of < 0.05 was considered statistically significant.

## Results

### Comparisons of general characteristics

**Fig. 1:**
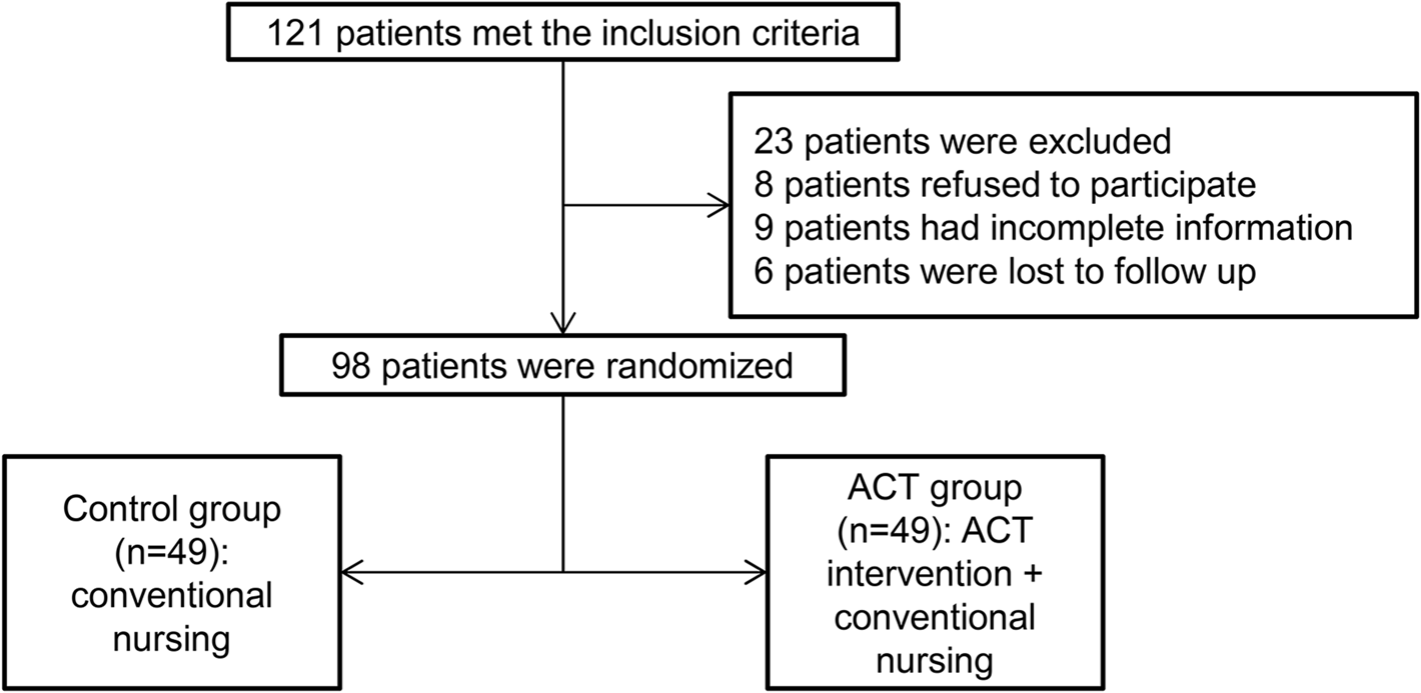
Flowchart of participant inclusion

A total of 122 young and middle-aged AMI patients that underwent PCI in our hospital between February 2019 and September 2019 met the inclusion criteria to be eligible for participation in our study; of these, 98 completed the intervention and the 3-month follow-up. The patients were randomized to the ACT or control group (*n* = 49 each). Figure 1 illustrates the flowchart of patient selection and allocation to groups. Table 2 shows the general characteristics of all patients in the trial. The mean age of the patients was 50.72 ± 5.91 years. The control group had 33 men and 16 women, and the ACT group had 35 men and 14 women. Furthermore, 94% and 91.8% of the patients in the control and ACT groups were married, respectively. Most of the patients were educated up to senior middle school or technical secondary school (42.9% in the control group vs. 32.2% in the ACT group). The baseline characteristics, including age, sex, marital status, educational level, number of children, treatment methods, income, and medical insurance types, did not significantly differ between the two groups (*P* > 0.05), suggesting that the two groups were comparable (Table 2).

**Table 2:** General characteristics of the patients in two groups

	Control group, *N* = 49	ACT group, *N* = 49
Age (years)	51.47 ± 6.10	49.98 ± 5.68
Sex		
Male	33	35
Female	16	14
Marital status		
Married	46	45
Divorced	1	1
Widowed	2	3
Educational level		
Junior middle school or lower	17	15
Senior middle school or technical secondary school	21	19
College or higher	11	15
Number of children		
0	1	0
1–2	37	38
≥ 3	11	11
Number of stents implanted		
1	29	32
2	20	16
≥ 3	0	1
Family income per person (yuan)		
≤ 3000	16	15
≤ 5000	26	28
> 5000	7	6
Payment		
Self-paying	4	3
Medical insurance for urban employees	24	26
The new rural cooperative medical care system	21	20

### Comparisons of psychological resilience before and after intervention in the two groups

Table 3 showed that the psychological resilience scores before the intervention did not significantly differ between the two groups (125.8 ± 25.9 vs. 127.1 ± 23.7, *P* = 0.789). At 3 months after the intervention, the ACT group had significantly higher psychological resilience scores than the control group (221.1 ± 17.3 vs. 196.5 ± 19.3, *P* < 0.001), and this difference was also significantly higher than the level before the intervention in the ACT group (221.1 ± 17.3 vs. 125.8 ± 25.9, *P* < 0.001).

**Table 3:** Comparisons of the psychological resilience before and after intervention in the two groups

Group	Number	Total score of psychological resilience		*P*
		Before intervention	Three months after the intervention	
ACT group	49	125.76 ± 25.98	221.05 ± 17.33	< 0.001^a^
Control group	49	127.11 ± 23.65	196.54 ± 19.26	< 0.001^a^
*P*		0.789^b^	< 0.001^b^	

^a^comparison before and after intervention between the patients in the same group; ^b^comparison between the twogroups at the same time

### Comparisons of self-management skills of the patients in the two groups

The self-management skills did not significantly differ between the two groups at the pre-intervention measurement (*P* > 0.05, Table 4). The scores of seven dimensions (health belief, diet management, disease coping, exercise management, environment management, and resource utilization) were significantly higher in the ACT group than in the control group after the intervention (all *P* < 0.05). These scores were also significantly higher at the post-intervention measurement than before the intervention in the ACT group (*P* < 0.05).

**Table 4:** Comparisons of self-management skills of the patients in the two groups

Dimension	Before intervention		*P*_1_	Three months after the intervention		*P*_2_
	ACT group (*n* = 49)	Control group (*n* = 49)		ACT group (*n* = 49)	Control group (*n* = 49)	
Health belief	23.34 ± 9.36	24.61 ± 10.10	0.520	38.75 ± 4.84	32.66 ± 6.59	< 0.001
Diet management	15.21 ± 5.77	14.76 ± 7.32	0.736	21.67 ± 2.60	18.44 ± 4.75	< 0.001
Disease coping	12.14 ± 3.88	11.53 ± 4.77	0.489	17.45 ± 1.59	14.71 ± 3.64	< 0.001
Exercise management	13.80 ± 6.15	14.34 ± 6.39	0.671	21.07 ± 2.78	19.12 ± 3.47	0.003
Environment management	14.31 ± 7.18	15.45 ± 6.61	0.415	20.22 ± 2.85	17.39 ± 4.25	< 0.001
Resource utilization	15.74 ± 5.92	14.87 ± 6.46	0.489	20.30 ± 2.19	18.11 ± 4.50	0.003
Self-efficiency	14.75 ± 6.46	15.34 ± 6.29	0.648	21.35 ± 2.41	17.62 ± 4.88	< 0.001

*P*_1_ comparisons between the two groups before the intervention, *P*_2_ comparisons between the two groups after the intervention

The primary outcome of this study was the psychological resilience of the patients in the two groups at 3 months after the intervention. A total of 49 patients who met the inclusion and exclusion criteria were included in the ACT group, and another 49 patients were included in the control group. The mean psychological resilience of patients in the ACT group at 3 months after the intervention was 221.05 ± 17.33 points, while that of the control group at 3 months after the intervention was 196.54 ± 19.26 points. The probability of the type I error (*α*) was set at 0.05. Then, the numbers of patients, means, and standard divisions in the two groups were input into the software, and the results revealed a statistical power of 1.00, suggesting that the results of this study were reliable.

## Discussion

ACT is an intervention that enhances well-being and reduces distress by increasing psychological flexibility and resilience. Rather than trying to control what a person thinks or feels, ACT helps individuals change their relationship with the events that have transpired [11, 12]. In the ACT, the health behavior interventions do not explicitly involve replacing previous unhealthy psychological events with newer healthy events; instead, ACT inculcates acceptance of the unhealthy psychological events [10]. Thus far, no study has investigated how ACT affects self-management and psychological resilience in young and middle-aged patients after PCI for AMI. Therefore, we studied the effect of ACT in psychological resilience in young and middle-aged AMI patients who underwent PCI.

ACT is well known for its positive effects in patients with chronic conditions. It has been used since its inception not only to treat patients with mental health disorders but also to help patients with physical, behavioral, and other issues cope with their conditions. Psychological resilience is the process of adaptation in the face of severe threats and high-risk environments, and an improvement in an individual’s psychological resilience could well promote both his and her physical and psychological health [13, 14]. Some studies suggested that resilience was defined by certain characteristics that enable individuals to thrive when they encounter adversity; these include hope (i.e., positive readiness and expectancy), motivation, optimism, sense of coherence (i.e., recognizing the world as meaningful and predictable), preexisting social support, and spirituality [15, 16]. Moreover, many studies have reported that psychological resilience was the key to coping with and overcoming chronic illness, reducing emotional distress, and improving self-perception [17, 18].

MI in young and middle-aged patients is a major traumatic event, and it could induce various psychological stresses [19]. Therefore, we performed an active four-stage intervention based on different psychological adaptation processes in our study participants. Our findings showed that before the intervention, both groups had relatively low resilience, and there was no significant difference in the pre-intervention psychological resilience of the two groups. The psychological resilience scores significantly increased in the ACT group after the intervention, and the scores were also significantly higher than the post-intervention scores in the control group, suggesting that psychological resilience was not a changeless psychological trait, and effective nursing intervention could promote the adaptation to stress among young and middle-aged AMI patients by improving their psychological resilience. Prevedini et al. [20] demonstrated that ACT made contributions to the treatment of patients with chronic physical diseases. Some reports showed that ACT could effectively improve the acceptance of patients with advanced cancer and also decrease cancer patients with the psychological burden [21, 22]. These findings support our results, suggesting that ACT could be applied in young and middle-aged patients who undergo PCI for primary MI, which substantially improves psychological resilience. The aim of ACT is not to change the physical and mental conditions but to change the attitude of the patients regarding their condition, helping them accept the disease status quo, establish a positive lifestyle and ideology, improve the level of psychological resilience, affirm self-value (self-efficacy), and strengthen self-management ability.

AMI is an acute disease with sudden onset and severe conditions. Thus, the patient believes that the disease is very serious, and the treatment risk is very high, inducing negative emotions such as low anticipation in patients [23]. Often, young and middle-aged patients with AMI are in the rising phase of their careers and serve as financial and spiritual support systems for their families. Not only can AMI reduce their social functions, but it can also turn them from caregivers to care recipients. The stimulations from the disease and worries about the risks of the operation and its outcomes can reduce patient anticipation and alter their attitudes to the present and future, as well as the postoperative self-management skills. Patients with high self-management skills are more active, with higher effort levels and better persistence regarding implementing health promotion behaviors; thus, they have a better quality of life [24]. In this study, the scores of the seven dimensions of self-management skills were relatively low in both groups at the pre-intervention assessment, and the scores did not significantly differ between the groups. This result could be associated with insufficient disease-related knowledge and inadequate attention on the disease after the first episode of AMI, as well as the educational levels and incomes of the patients. Increasing self-management skills and rebuilding the life beliefs of the patients is a low-cost and effective disease management method. The findings demonstrated that after standardized intervention with four-stage ACT, the scores of all seven dimensions were significantly higher in the ACT group than in the control group, and the post-intervention score of the ACT group also significantly increased from the pre-intervention score. These findings suggested that ACT could improve the self-management skills and promote the recovery of young and middle-aged patients who undergo PCI for primary MI.

Our study had some limitations. First, as this was a pilot randomized controlled trial, the sample size was not calculated and might be small, which could result in inducing bias in the results. Still, the statistical power was calculated as 1.00 finally, which suggests that this study’s results were reliable. Second, the observation time of 3 months after the intervention was relatively short, and the long-term benefits of ACT could not be studied. Therefore, more multicenter randomized controlled studies with larger sample sizes are needed, which could provide a more accurate picture of the psychological effects of ACT on young and middle-aged AMI patients.

### Limitations

It is of great practical importance to analyze the effect of ACT therapy on negative emotions such as anxiety and depression, and other psychological problems in young and middle-aged patients after PCI for first myocardial infarction. Still, anxiety and depression were not included in this study. It is, therefore, necessary to conduct a multicenter, large sample prospective study that will include more psychological evaluation indicators such as anxiety and depression.

## Conclusions

In summary, the results of our study revealed that ACT could improve psychological resilience, reduce negative emotions, increase self-efficiency, enhance self-management skills in young and middle-aged AMI patients undergoing PCI, and thus substantially help improve the disease conditions.

## Data Availability

The datasets used and/or analyzed during the current study are available from the corresponding author on reasonable request.

## Abbreviations

AMI: Acute myocardial infarction
PCI: Percutaneous transluminal coronary intervention
ACT: Acceptance and commitment therapy
